# Rationale and design for SHAREHD: a quality improvement collaborative to scale up Shared Haemodialysis Care for patients on centre based haemodialysis

**DOI:** 10.1186/s12882-017-0748-6

**Published:** 2017-11-24

**Authors:** James Fotheringham, Tania Barnes, Louese Dunn, Sonia Lee, Steven Ariss, Tracey Young, Stephen J. Walters, Paul Laboi, Andy Henwood, Rachel Gair, Martin Wilkie

**Affiliations:** 1 Sheffield Teaching Hospitals NHS Foundation Trust, S5 7AU, Sheffield, UK; 2 University of Sheffield & NIHR CLAHRC YH, Sheffield, UK; 3 York Teaching Hospital NHS Foundation Trust, York, UK; 4 UK Renal Registry, Bristol, UK

**Keywords:** Randomised stepped-wedge, Breakthrough series collaborative, Patient activation, Supported self-care, Shared Haemodialysis Care

## Abstract

**Background:**

The study objective is to assess the effectiveness and economic impact of a structured programme to support patient involvement in centre-based haemodialysis and to understand what works for whom in what circumstances and why. It implements a program of Shared Haemodialysis Care (SHC) that aims to improve experience and outcomes for those who are treated with centre-based haemodialysis, and give more patients the confidence to dialyse independently both at centres and at home.

**Methods/Design:**

The 24 month mixed methods cohort evaluation of 600 prevalent centre based HD patients is nested within a 30 month quality improvement program that aims to scale up SHC at 12 dialysis centres across England. SHC describes an intervention where patients who receive centre-based haemodialysis are given the opportunity to learn, engage with and undertake tasks associated with their treatment.

Following a 6-month set up period, a phased implementation programme is initiated across 12 dialysis units using a randomised stepped wedge design with 6 centres participating in each of 2 steps, each lasting 6 months. The intervention utilises quality improvement methodologies involving rapid tests of change to determine the most appropriate mechanisms for implementation in the context of a learning collaborative. Running parallel with the stepped wedge intervention is a mixed methods cohort evaluation that employs patient questionnaires and interviews, and will link with routinely collected data at the end of the study period. The primary outcome measure is the number of patients performing at least 5 dialysis-related tasks collected using 3 monthly questionnaires. Secondary outcomes measures include: the number of people choosing to perform home haemodialysis or dialyse independently in-centre by the end of the study period; end-user recommendation; home dialysis establishment delay; staff impact and confidence; hospitalisation; infection and health economics.

**Discussion:**

The results from this study will provide evidence of impact of SHC, barriers to patient and centre level adoption and inform development of future interventions to support its implementation.

**Trial registration:**

ISRCTN Number: 93999549, (retrospectively registered 1^st^ May 2017); NIHR Research Portfolio: 31566

## Background

In the UK approximately 23,000 people attend in-centre facilities three times a week to receive haemodialysis (HD) [1]. Structural arrangements in dialysis units tend to create an environment in which patients become passive recipients of their care, engaging little with their own treatment. There is considerable evidence of the benefits of supported self-care in long term conditions [2, 3]. Low health literacy amongst dialysis patients is associated with worse survival [4] whereas self-motivation and patient education results in preferable clinical parameters e.g. phosphate control [5] and fluid balance [6]. Dialysis services are experiencing considerable pressure to deliver high quality care in the face of fiscal challenge, often when approaching capacity. An important mechanism to ensure that quality of care is maintained is to engage service users as true partners in their own care; self-management is an ambition in “Kidney Health: Delivering Excellence” [7]. The tradition of self-care extends to the 1,113 UK patients who performed haemodialysis at home in 2014 [1]. In addition to the better survival [8] and quality of life [9] associated with home HD (HHD), increasing HHD from 4.1% in the UK to the 12.9% used in Australia and New Zealand [10] would lead to estimated annual savings to the UK National Health Service of £16M (an increase of 1400 people treated with HHD at a saving of £12000 per patient per year [11]).

SHC describes an intervention where people treated with in-centre HD are given the opportunity to learn tasks relating to their own dialysis treatment. HD treatment is standardised requiring a number of key steps for its preparation, delivery and discontinuation. For SHC to be adopted consistently across dialysis services several changes become necessary. Health care professionals require a change in their roles from one in which they undertake repetitive tasks for patients to one where they become educators and facilitators in order to support patients to take a greater role in their own care. Patients require encouragement to change their expectation of care to one where they become involved in their own treatment. This approach gives centre based dialysis patients access to the benefits of participating in their own care while increasing opportunities for home dialysis.

## Methods/Design

### Aim

This study aims to assess the effectiveness and economic impact of a structured programme to encourage patient involvement in centre-based haemodialysis (HD), and to understand what works for whom in what circumstances and why. This supported self-care intervention is intended to improve experience and outcomes for those who are treated with centre-based haemodialysis, and give more patients the confidence to dialyse independently both at centres and at home.

### Study design and timeline

**Fig. 1:**
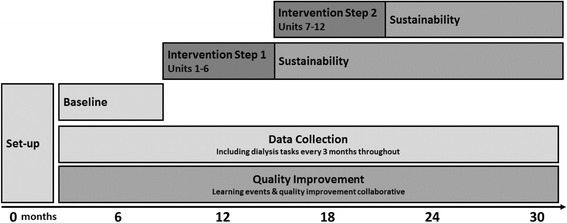
Stepped wedge evaluation of quality improvement to facilitate shared haemodialysis care

End points will be quantified following a stepped-wedge introduction of the intervention, in which 6 centres are randomly allocated to start implementation in step 1, with the remaining 6 starting in step 2. Each step lasts 6 months, and including the baseline phase the study lasts 24 months. The study phases are outlined in Fig. 1 and the schedule of instruments in Table 1. The programme entered the baseline phase in October 2016.

**Table 1:** Data collection instruments

Instrument	Frequency	Content
Demography form	Once, at start	Ethnicity, educational level, home and employment circumstances (adapted from [27])
Tasks questionnaire	3 monthly	15 dialysis related tasks (Table 2)
Think Kidneys questionnaire	6 monthly	POS-S Renal symptom score [16], quality of life (EQ-5D-5L [15]), patient activation measure [14]
SHAREHD uptake indicators form	6 monthly	Meta-cognition (memory & concentration [28]), Health literacy [29], dialysis access and attitudes to self-needling
Health economic form	6 monthly	Transport, dependants, arrangements made and duration of attendance for patient and companions who also attend dialysis
Status change form	Event Driven	Transplantation, mortality, changes in dialysis modality & location

### Study setting and participants

The objective is to recruit 600 prevalent in-centre HD patients from the 12 participating renal centres during the baseline phase. All suitable prevalent dialysis patients at selected centres are entered into a screening log and from this patients are approached and given the opportunity to participate in the research with the intention of recruiting up to 50 patients at each site. Once eligible patients have been given study information, informed consent is taken by trained delegated members of the local research team at a subsequent dialysis session. This includes consent for data linkage to hospital episode and UK Renal Registry data. Participants are also given the opportunity to be involved in interviews and workshops conducted by the evaluation team. As the study is questionnaire based we anticipate that more than 90% of approached patients will agree to be recruited to the study.

### Inclusion/exclusion criteria

Inclusion criteria are for patients to be established on centre based HD and have capacity to give written informed consent. Exclusion criteria are those who are too unwell to engage in the study as judged by the clinical team or unable to understand written and verbal communication in English.

### Intervention

The intervention at the centre of this study is that centre based HD patients are given the choice to undertake treatment related tasks, listed in Table 2. These range from performing observations, preparatory hygiene, setting up the dialysis machine, securing dialysis access, programming the machine, initiating monitoring and discontinuing/completing dialysis.

**Table 2:** Shared Haemodialysis Care treatment tasks (note some tasks are mutually exclusive)

Patient preparation	Machine Preparation & Dialysis Initiation	During and after dialysis
Measuring your weight	Lining your dialysis machine	Responding to your machine alarms
Measuring your blood pressure and pulse	Priming your dialysis machine	Disconnecting the lines and completing your dialysis
Measuring your temperature	Preparing your dressing pack	Applying pressure to your needle sites OR Locking your own tunnelled line
Washing your hands prior	Programming your dialysis machine	Giving your own anaemia injections (such as epoetin)
	Needling your fistula/graft OR Preparing your tunnelled line	
	Connecting the lines to your fistula/graft/tunnelled line and commencing dialysis	

A series of learning events are planned with teams comprising approximately five individuals from each participating site. These include clinicians, nursing staff, patient partners and additional relevant personnel (e.g. psychologist, service managers as determined by individual sites). The learning events are designed to review the objectives of SHC, share patient and clinician experience, teach and review quality improvement methodologies, and develop rapid tests of change to optimise local implementation. Learning is underpinned through the SHC website [12], teleconferences, social media platforms and newsletters, while staff education is supported by a bespoke nursing training course developed during pilot work [13]. A patient advisory group has been established in order to support their involvement and ensure that the programme maintains patient focussed objectives.

### Outcome measures and quantitative data collection

Efficacy Endpoints - The primary binary outcome is a change in the proportion of sampled HD patients completing 5 or more out of 14 tasks (Table 2). The secondary binary outcome is an absolute increase HHD and in centre independent dialysis of 4% within participating centres.

Quality and Safety Endpoints - Changes in patient activation [14]; quality of life (EQ-5D-5L) [15]; POS-S Renal symptom score [16] will be assessed using the “Your Health Survey” developed as part of the NHS England supported Transforming Participation in Chronic Kidney Disease program [17]. Responses to this survey will be transferred to the UK Renal Registry and uploaded to PatientView [18] for participating patients to review themselves and to be used to inform clinical consultations. Hospitalisation (all-cause and cause specific) will inform cost, benefits and harms assessments. The economic evaluation will take an NHS and social care perspective and will compare SHC with usual care in a cost-utility analysis based on a cost-per quality adjusted life years (QALYs) approach. The EQ-5D-5L will be used to measure health related quality of life at baseline, six and 12 months. Resource use will be estimated from a range of sources including an adapted cost questionnaire [19] and non-participant observation to quantify time spent performing HD tasks by patients and staff.

Hospital Episode Summary data will be used for information on comorbidity at the time of recruitment (by reviewing diagnosis codes from admissions up to 5 years prior to the start of the SHAREHD programme), hospitalisation (all cause and cause specific) before and after the implementation of SHAREHD to assess for benefits and harms, and inform health economic analyses. Similar datasets and diagnostic codes have been used to identify harms from hospital and home-based haemodialysis therapies and assess cost [20].

### Qualitative investigation and realist evaluation

Realist evaluation will be conducted complementary to the quantitative primary and secondary endpoints to determine if and how SHC works, for whom and in what context. Building on existing theories gained from literature, key stakeholders and the Yorkshire SHC pilot, a logic model will be constructed. This model will be developed throughout the project, and used to refine and test specific relevant hypotheses. Key contextual service characteristics will be determined across the units to further understand the linkage between variability in context, implementation and outcomes, and to inform interview sampling strategies. Semi-structured interviews will be conducted before and after the intervention with approximately 24 patients and 24 members of staff across participating sites. Programme theory and expert opinion will inform a stakeholder map from which key individuals will be selected for interview.

### Allocation and blinding

The study statistician (SJW) used computer generated random numbers to produce a random allocation sequence for the 12 dialysis centres; with 6 units randomised to intervention in the first step and the remaining 6 units randomised to the intervention in the step two. There was no patient-level stratification. The study manager (SL) was told of the random allocation sequence and then informed the 12 dialysis centres when they would be receiving the SHAREHD intervention. Individual trial participants at the 12 dialysis centres are recruited and enrolled by local clinical research network nursing staff. The study participants, centres and research staff are unblinded.

### Sample size calculation

Assuming that the baseline level of completing 5 tasks is around 15%, an ICC of 0.05 and an average cluster size of 25 HD patients; then using the STATA stepped wedge command [21] with a stepped wedge design of 3 steps (including baseline) and 12 clusters, with 6 clusters randomised at each step, we will have 90% power to detect an increase in the event rate from 15% to 30% as statistically significant at the 5% two-sided level. If we assume that the baseline level of the secondary outcome measure of HHD is around 2% in participating clusters an ICC of 0.05 and an average cluster size of 25 HD patients; then with a stepped wedge design of 3 steps (including baseline) and 12 clusters, with 6 clusters randomised at each step will we have 80% power to detect an increase in the event rate from 2% to 7.2% as statistically significant at the 5% two-sided level. In recognition of a mortality rate of 17% per annum [20], a background renal transplantation rate and to mitigate the risk of incomplete data collection, the target recruitment per participating site was increased to 50.

### Data collection

Paper questionnaires will be batched, and securely transferred to the Sponsor site (Sheffield Teaching Hospitals NHS Foundation Trust)^1^ to be entered into the research database and will be retained securely for audit purposes. Interview audio recordings will encrypted, pseudo-anonymised and be destroyed after transcription.

### Statistical analysis plan

The primary and secondary outcomes will be compared across the intervention and control clusters using a longitudinal random (or multi-level mixed) effects logistic regression model (with time, phase or step), group (intervention or control) and individual patient characteristics such as age and gender as covariates; and the renal unit or cluster as a random effect. These models will take into account the clustering of outcomes by units. The odds ratio estimate for the intervention effect and its associated confidence interval will be reported from the model. Statistical associations between patient characteristics, dialysis schedules and outcomes will also be explored.

### Monitoring

The quality improvement initiative and cohort study is monitored through a project board, evaluation and patient advisory groups. The evaluation board monitors the progress of the study including data completeness and safety issues.

## Discussion

The objective of this evaluation is to better understand the impact of participating in treatment related tasks among people who receive dialysis at treatment centres. The outcomes that we will examine through this study include access to home or independent dialysis in centre, quality of life, symptom scores, as well as harms including infection and hospitalisation. The intervention at the heart of this protocol is to create an environment where dialysis nurses consistently give patients the opportunity to choose to learn and undertake treatment related tasks. It is our plan that this will be implemented at participating sites through a series of workshops based on quality improvement methodologies, nested in a structured collaborative in which teams from participating sites are able to share learning in order to identify the most effective approaches.

Patient involvement in health care comes in a variety of forms including engaging people to keep healthy, shared decision making, choosing a provider and self-management support [22]. The evolution of person-centred care has progressed over several decades contributed to by a succession of key documents, including most recently the NHS five year forward view [23]. Patient training is central to the provision of dialysis home therapies in order to provide individuals with the required skills necessary to manage their condition in the community away from the hospital services. Alternatively those who receive their dialysis treatment at centres are much less likely to be engaged in their own treatment. There is robust evidence that informing patients about their condition and providing educational opportunities for them to engage in their own care leads to improved outcomes [2]. Current arrangements limit opportunities for centre-based dialysis patients to take a significant role in improving their own outcomes.As a consequence, the existing approach is potentially disadvantageous to centre based HD patients and could be challenged on the basis of equity, since those who take up HHD tend to have lower deprivation scores and are more likely to be Caucasian.

The strengths of our protocol include the stepped wedge design with a random allocation to reduce the risk of bias, particularly relevant in a quality improvement intervention. We selected instruments on the basis of clear evidence of their utility. The Patient Activation Measure (PAM) [24] is being used under licence from NHS England to explore factors that impact on readiness to participate in dialysis related tasks. Low patient activation is associated with a range of poorer healthcare outcomes including readmission to hospital, medical errors and loss of confidence in healthcare providers [25]. The questions on mobility, symptom burden, cognition, health literacy and attitudes to self-needing included in our questionnaires (Table 1) were identified though our pilot work as potentially impacting on the individual’s ability to participate in HD tasks. Identifying barriers to involvement is clearly important particularly as the SHC concept is that patients are supported to take on as much or as little as they feel able to do. A small amount of engagement is meaningfully different from an environment where participation is discouraged. The duration of the study is sufficient to assess whether perceived benefits to patients are sustained or whether the repetitive nature of dialysis treatment results in a loss of enthusiasm for engagement over time. Patient partners are central in the design of the intervention, selection of endpoints and instruments and ensuring outputs remain relevant to the end-user (co-production).

The challenges of the adopted approach include competing pressures that health care professionals face in their day to day work and that the heterogeneity of HD units may limit the effectiveness of the intervention. To address these concerns we have adopted quality improvement methodologies that are intentionally tailored to support local configuration. Participant drop-out during the course of the 24 month protocol has been mitigated by doubling the initial cohort size above that required by the power calculation.

### Summary

This prospective 12 site cohort study sets out to relate the impact of learning treatment related tasks for people who receive centre-based dialysis. The objective is to support a cultural change where people treated with dialysis are encouraged to become active partners in their care irrespective of the location of their treatment. The instruments and outcome measures have been selected because of their relevance to evaluating the barriers and drivers of supported self-care in the centre based HD environment. The stepped-wedge design is underpinned by intervention delivery within a quality improvement collaborative. Clearly that approach requires sensitivity to the abilities and preferences of patients while creating an environment where participation is normalised.

## Abbreviations

EQ-5D-5L: European quality of life (QoL) 5 dimension 5 level score
HD: Haemodialysis
ICC: Intraclass correlation coefficient
PAM: Patient activation measure
QALY: Quality-adjusted life years
SHC: Shared Haemodialysis Care

## Footnotes

1 Sheffield Teaching Hospitals NHS Foundation Trust, R & D Contact Dr Dipak Patel, Research Manager, Clinical Research Office Sheffield Sheffield Teaching Hospitals NHS Foundation Trust D Floor, Royal Hallamshire Hospital Glossop Road, Sheffield S10 2JF Dipak.Patel@sth.nhs.uk T +44 (0) 114 2265941 Fax. +44 (0) 114 2265937
